# Effect of Recombinant Human Growth Hormone (rhGH) Use on Genetic Methylation Patterns and Their Relationship with Body Composition in Small-for-Gestational-Age (SGA) Newborns

**DOI:** 10.3390/biomedicines13061288

**Published:** 2025-05-23

**Authors:** Juan M. Alfaro Velásquez, Elsa Maria Vásquez Trespalacios, Rodrigo Urrego, María C. Arroyave Toro, María del Pilar Montilla Velásquez, Cecilia Maria Díaz Soto, Juan C. Zuluaga Vélez, Verónica Jaramillo Henríquez, Jorge Emilio Salazar Flórez, Fernando P. Monroy, Hernando Alirio Palacio Mosquera, Sara Vélez Gómez, Ronald Guillermo Pelaez Sánchez

**Affiliations:** 1 Life and Health Sciences Research Group, Graduate School, CES University, Medellin 050021, Colombia; juan.alfaro@udea.edu.co (J.M.A.V.); hernando.palacio1@ces.edu.co (H.A.P.M.); savelezg@uces.edu.co (S.V.G.); 2 Medical Surgical Specialties Group, Faculty of Medicine, CES University, Medellin 050021, Colombia; evasquez@ces.edu.co; 3 INCA-CES Group, School of Veterinary Medicine and Zootechnic, CES University, Medellin 050021, Colombia; rurrego@ces.edu.co; 4 Pediaciencias Group, Universidad de Antioquia, Medellin 050010, Colombia; maria.arroyavet@udea.edu.co (M.C.A.T.); maria.montilla@udea.edu.co (M.d.P.M.V.); 5 Observatos Group, Tecnológico de Antioquia, Medellín 050034, Colombia; cecilia.diaz@tdea.edu.co; 6 Special Care Unit, Clínica CES, CES University, Medellín 050021, Colombia; juanzuluaga@clinicaces.edu.co; 7 GEINCRO Research Group, School of Health Sciences, San Martín University, Sabaneta 055457, Colombia; veronica.jaramillo@sanmartin.edu.co (V.J.H.); jorge.salazarf@sanmartin.edu.co (J.E.S.F.); 8 Department of Biological Sciences, Northern Arizona University, Flagstaff Arizona, AZ 86011, USA; fernando.monroy@nau.edu

**Keywords:** SGA newborns, rhGH, body composition, genes, methylation, epigenetics

## Abstract

**Background:** Low birth weight in newborns is of multifactorial origin (fetal, maternal, placental, and environmental factors), and in one-third of cases, the cause is of unknown origin, with high infant morbidity and mortality. The main treatment for regaining weight and height in children with low birth weight is the application of growth hormones. However, their role as a protective factor to prevent an increase in body composition and the development of metabolic diseases is still poorly understood. **Methodology**: A case–control study was conducted in a cohort of patients consulted at the CES Pediatric Endocrinology Clinic, Medellín, Colombia, between 2008 and 2018. We evaluated sociodemographic and clinical variables. Additionally, the identification of differential patterns of genomic methylation between cases (treated with growth hormone) and controls (without growth hormone treatment) was performed. The groups were compared using Fisher’s exact test for qualitative variables and Student’s *t*-test for the difference in means in independent samples. The correlation was evaluated with the Pearson coefficient. **Results:** Regarding clinical manifestations, body mass index (BMI) was higher in children who did not receive growth hormone treatment, higher doses of growth hormone treatment helped reduce body mass index (R: −0.21, and *p* = 0.067), and the use of growth hormone was related to a decrease in triglyceride blood concentrations (*p* = 0.06); these results tended towards significance. Regarding genome-wide methylation patterns, the following genes were found to be hypermethylated: *MDGA1, HOXA5, LINC01168, ZFYVE19, ASAH1, MYH15, DNAJC17, PAMR1, MROCKI, CNDP2, CBY2, ZADH2, HOOK2, C9orf129, NXPH2, OSCP1, ZMIZ2, RUNX1, PTPRS, TEX26, EIF2A4K, MYO1F, C2orf69*, and *ZSCAN1*. Meanwhile, the following genes were found hypomethylated: *C10orf71-AS1, ZDHHC13, RPL17, EMC4, RPRD2, OBSCN-AS1, ZNF714, MUC4, SUGT1P4, TRIM38, C3, SPON1, NGF-AS1, CCSER2, P2RX2, LOC284379, GGTA1, NLRP5, OR51A4, HLA-H*, and *TTLL8*. **Conclusions:** Using growth hormone as a treatment in SGA newborns helps regain weight and height. Additionally, it could be a protective factor against the increase in adolescent body composition.

## 1. Introduction

Low birth weight in newborns arises from multiple factors, including fetal, maternal, placental, and environmental influences. In about one-third of cases, the cause remains unknown, leading to high rates of infant morbidity and mortality. The most widely accepted definition of small for gestational age (SGA) is a newborn with weight and/or height less than or equal to two standard deviations from the mean of a reference population by sex and gestational age [1]. On the other hand, for the World Health Organization (WHO), any child weighing less than 2500 g at birth is small for gestational age (SGA) [2]. This medical condition is a public health problem with immediate and long-term implications for the newborn’s health. Due to its high prevalence in developing countries, newborns with this condition are subject to continuous medical monitoring. They are at increased risk for various health problems derived from the increase in the fat mass/lean mass ratio and the tendency to develop metabolic syndrome, neurological developmental delay, cardiovascular disease, impaired glucose tolerance, insulin resistance, obesity and increased abdominal circumference, growth delay, motor development problems, and other health concerns [3,4,5,6,7].

It is estimated that worldwide, between 15% and 20% of newborns have low birth weight, which represents more than 20 million newborns per year [2]. Until 2020, the prevalence of children with low birth weight in Latin America was 9.6%, in Colombia 11% [8]. In the Antioquia Department, the prevalence in 2022 was reported to be between 9.8% and 13.3% [9]. In 1985, the use of recombinant human growth hormone (rhGH) was approved in children with growth hormone deficiencies. This treatment has been shown to induce rapid changes in body composition, including reduced levels of the protein Leptin, decreased visceral fat, enhanced lipolytic activity, and modest increases in adiponectin protein concentrations [10,11,12,13]. In addition, in 2003, the use of rhGH was approved for the treatment of the SGA population by the FDA (Food and Drug Administration) and EMA (European Medicines Agency) in SGA children with persistent short stature to two years of age [14]. This hormone is related to regulating human body growth and helps SGA children in regaining their height and weight. Additionally, it influences carbohydrate metabolism, fat metabolism, and body composition [15,16,17,18]. It is believed that rhGH is a protective factor for SGA children not to develop obesity in adulthood, since various studies show that rhGH has an inverse relationship with fat mass levels and body mass index (BMI) [15]. Obesity is a complex condition arising from the interplay of multiple genes and environmental factors [16]. Genes associated with the etiology of obesity include genes encoding peptides aimed at transmitting hunger and satiety signals and signaling pathways involved in the growth and differentiation of adipocytes and genes related to controlling energy expenditure [17]. The obesity map established in 2000 suggests the existence of monogenic obesity, Mendelian-inherited obesity, and polygenic obesity (associated with multiple genes) [17]. Currently, there is solid scientific evidence indicating that there are around 71 genes that potentially induce the appearance of obesity; 15 of these are linked to increased body fat volume [17].

Numerous studies have demonstrated that epigenetic regulatory mechanisms play a significant role in the onset and progression of obesity and its associated metabolic complications. The environment modulates the individual’s epigenetic mechanisms, nutritional factors, and physiological conditions [18]. Several genes have been described whose methylation is involved in obesity, type 2 diabetes, and metabolic syndrome. These genes code for different signaling pathways of insulin resistance and secretion (*ATM, PTPRN2, PSMD10*, and *NSF*), adipogenesis (*SLC25A24* and *PAX8*), inflammatory processes (*TNFRSF8* and *SLIT3*), and mitochondrial processes (*PM20D1* and *LCLAT1*) [18]. Other studies analyzing methylation patterns between obese and non-obese individuals have shown that genes such as *NRF1*, *ADR1B*, and *PTPRN2* have differential methylation patterns, highlighting their potential as biomarkers to predict the risk of developing obesity [19,20]. Children born small for gestational age (SGA) often have a deficiency in growth hormone production [21]. To mitigate this deficiency, recombinant growth hormone (rhGH) has been used for decades, showing safe and positive outcomes in height and weight recovery, lipid metabolism, and blood pressure regulation, particularly when treatment begins at puberty [22]. Moreover, rhGH treatment has shown favorable changes in body composition, with an increase in lean mass and a decrease in fat mass [23,24]. The current research aims to investigate the effects of recombinant human growth hormone (rhGH) use on genetic methylation patterns and their relationship with body composition in small-for-gestational-age (SGA) newborns in Medellín, Colombia.

## 2. Materials and Methods

### 2.1. Study Population

The present study is a case–control study nested in a follow-up cohort. Cases are children born <2500 g using rhGH, and controls are children born <2500 g not using rhGH. The rhGH dose corresponds to the annual average (U/k/week–mg/kg/day). One hundred and eleven patients with SGA were recruited for the study from the pediatric endocrinology consultation of the CES clinic in Medellín city between 1 July 2008 and 30 June 2018, for 10 years of follow-up. Forty-seven SGA patients received treatment with recombinant growth hormone, and 65 were not treated. The following inclusion criteria were applied: children from the SGA cohort seen in the Pediatric Endocrinology clinic at CES Clinic in Medellin, Colombia, consent and assent accepted to participate in the study, clinical follow-up between 1 July 2008 and 30 June 2018, children born at less than <2500 g, children treated with growth hormone (cases), and children not treated with growth hormone (controls). Additionally, patients who met any of the following exclusion criteria were withdrawn from the study: congenital infectious diseases that alter birth weight (congenital rubella, congenital syphilis, herpes virus, STORCH microorganisms (toxoplasmosis, rubella, cytomegalovirus, herpes simplex, and HIV)), polymalformative conditions that alter birth weight, trisomy [13,18,21], Turner syndrome, growth hormone deficiency including hypopituitarism, SGA patients treated with rhGH for less than 2 years of continuity, patients with monogenic obesity at the time of assessing body composition, not agreeing to be included in the study, and/or not allowing the use of their data. After applying the exclusion criteria, the population was reduced to 28 participants: 14 patients treated with growth hormone (cases) and 14 untreated patients (controls), all assessed for all clinical and sociodemographic characteristics. From this population, 8 cases and 8 controls were chosen to evaluate the human methylation profiling using the Illumina Methylation EPIC Chip platform (Illumina, Inc., San Diego, CA, USA). In total, 16 patients were evaluated according to the economic resources of the project, and these were chosen at random from among the cases and controls.

### 2.2. Collection of Clinical and Sociodemographic Data

Clinical and sociodemographic data of patients were collected from records deposited in medical histories. The information was consolidated in a database built with Microsoft Excel software for Windows 11, considering the temporality of the records, verifying that the data are correctly filled out, and minimizing digitization errors. The following variables were analyzed: newborn with low birth weight (<2500 g), gestational diabetes (diabetes that appears for the first time during pregnancy), gestational hypertension (blood pressure ≥ 140/90 mmHg after 20 weeks of gestational age), and gestational hemorrhage (vaginal bleeding during pregnancy). Patients were weighed using a Seca^®^ 274 electronic scale (Hamburg, Germany), the abdominal circumference was measured with a Prodalia^®^Bogotá-Colombia measuring tape scaled in (1, ½, and ¼ centimeters), and height was determined using a Harpenden^®^ stadiometer (Reference: HAR.98602VR, Wales, UK). Body mass index (BMI) was calculated using the recorded weight and height data. Descriptive analysis was performed using central tendency and dispersion statistics for quantitative variables. For categorical variables, frequency and relative frequency analysis were performed. This information is presented in 2 × 2 contingency tables, and the level of comparison was performed using the Chi-Square significance test between patients treated with rhGH and untreated patients. For qualitative variables, comparisons between groups were made using Fisher’s exact test. In independent samples, Student’s t test was used (difference in means). The level of significance was *p* < 0.05 (95% CI). Correlation was assessed using Pearson’s coefficient.

### 2.3. Blood Biochemical Evaluation

Blood samples were taken from patients who had fasted for 8 h, and the blood sample was taken by phlebotomy of the antecubital fossa vein. The samples were processed by spectrophotometry in an analyzer (DxC700 AU, Beckman Coulter/serial B98654/Brea, CA, USA). Triglycerides, total cholesterol, high-density lipoprotein cholesterol (HDL-C), low-density lipoprotein cholesterol (LDL-C), and blood sugar were measured from these blood samples using an automated clinical chemistry analyzer BS 230 (Mindray, Medical International Limited, Shenzhen, China).

### 2.4. DNA Sample Extraction

DNA was extracted from 200 μL of peripheral blood obtained by venipuncture of the forearm of small-for-gestational-age (SGA) newborns. DNA extraction was performed using the QIAamp DNA Mini K kit (QIAGEN^®^, Hilden, Germany), which is based on the absorption of DNA through silica columns. The procedure followed the manufacturer’s instructions: blood cells were lysed by adding 20 μL of buffer QP (QIAGEN protease), 200 μL of the blood sample, and 200 μL of buffer AL (lysis buffer). The mixture was then vortexed for 15 s (Labnet Vortex Mixer, Edison, NJ, USA) and incubated for 10 min at 56 °C in a thermoblock (Miulab, Sku: Minic-100). Subsequently, 200 μL of ethanol was added, and the sample was vortexed for 15 s. The lysate was then transferred to a QIAamp-Mini spin column and centrifuged for 1 min at (6000× *g* or 8000 rpm) in a centrifuge (Thermo Scientific, MicroCL-17, Waltham, MA, USA). The spin column was then transferred to a new wash tube, and 500 μL of buffer AW1 (wash buffer 1) was added and centrifuged for 1 min at (6000× *g* or 8000 rpm). The spin column was then transferred to a new wash tube, and 500 μL of buffer AW2 (wash buffer 2) was added and centrifuged for 1 min at maximum speed (20,000× *g* or 14,000 rpm). The QIAamp-Mini spin column was then transferred to a new wash tube and centrifuged for 3 min at maximum speed (20,000× *g* or 14,000 rpm). The QIAamp-Mini spin column was then transferred to a new elution tube. An amount of 200 μL of buffer AE (elution buffer) was added and centrifuged for 3 min at maximum speed (20,000× *g* or 14,000 rpm).

### 2.5. Quantification and Evaluation of DNA Quality

DNA integrity was assessed using 1% agarose gels in an electrophoresis chamber (Kalstein, Paris, France) and a bioanalyzer (Agilent 2100, Santa Clara, CA, USA). DNA (5 μL) was immediately revealed by electrophoresis. As follows, 5 μL of the DNA reaction was mixed with 5 μL of running buffer and streaked onto a 1% agarose gel (Seakem Le agarose) together with 10 μL of a 1000 bp molecular weight marker (1000 bp GeneRulerTM DNA Ladder). For the electrophoretic run, 500 mL of 1X TAE buffer (40 mM Tris–Acetic Acid and 1 mM EDTA) was used as an ion source at a constant 90 volts for 40 min in a chamber (Kalstein, Paris, France). The gel was stained with 2 μL of ethidium bromide (5 mg/mL), and the amplification products were visualized on a Geldoc XR+ ultraviolet light transilluminator (BioRad, Hercules, CA, USA) and photographed with Quantity One software (BioRad, Hercules, CA, USA, Version 4.6).

### 2.6. Epigenetic Sequencing of Human Methylation Profiling

Bisulfite conversion of DNA was performed using the EZ DNA Methylation-DirectTM kit (Zymo Research, Freiburg, Germany) according to the manufacturer’s instructions. The extracted and purified DNA were converted using the CT conversion reagent provided by the kit and incubated at 98 °C for 8 min, followed by 64 °C for 3.5 h in a thermal cycler. After DNA conversion, unmethylated cytosines are transformed into uracil, while methylated cytosines remain unchanged. The converted DNA were washed and cleaned using the Zymo-Spin™ IC column and dissolved in 10 mL of M-Elution Buffer.

Human methylation profiling was carried out on 16 small-gestational-age (SGA) newborns through the Illumina Methylation EPIC Chip platform (Illumina, Inc., San Diego, CA, USA). This product includes inbuilt 935,000 CpG regions in enhancer regions, gene bodies, promoters, and CpG islands. The 16 samples met the following characteristics: DNA concentration (>1.4 ug DNA genomic), purity (>1.5), and concentration (>70 ng/μL, non-degraded, non-amplified). The following analyses were subsequently performed on the results of epigenomic sequencing: image analysis, extract raw data, data pre-processing, and quality check, filtering differentially methylated probes between comparison samples (delta mean, linear regression, odds ratio of M value, and fold change). Subsequently, the differentially methylated region (DMR), hierarchical clustering (Euclidean method and complete linkage), Gene Enrichment, and Functional Annotation Analysis were performed. Each methylation data point is represented by fluorescent signals from the M (methylated) and U (unmethylated) alleles. Background intensity computed from negative controls was subtracted from each analytical data point. The ratio of fluorescent signals was then computed from the two alleles ß = (max(M, 0))/(|U| + |M| + 100). The ß-value reflects the methylation level of each CpG site. A ß-value of 0 to 1 signified percent methylation from 0% to 100%, respectively, for each CpG site. The results were analyzed using the following statistical methods: delta mean, odds ratio, fold change, and linear regression. All analyses were performed using the software R (4.4.1 version) and R-Studio Desktop (2024.09.0 + 375 version).

### 2.7. Bioinformatic Prediction of Protein Interactions

The STRING web server studied the interaction of proteins with differential methylation patterns (https://string-db.org, accessed on 1 December 2024). STRING predicts the main proteins interacting with the query gene based on gene fusion, co-expression, function, and experimental data. It shows combined scores for each interacting protein, ranging from 0 to 1, where 0 shows the lowest interaction and 1 indicates the highest interaction.

### 2.8. Structural Modeling and Functional Bioinformatics Analysis

The InterProScan web server was used to identify the domains, biological processes, molecular function, and cellular components of the protein (https://www.ebi.ac.uk/interpro, accessed on 1 December 2024). The three-dimensional structure of the protein was modeled using the SWISS-MODEL web server (https://swissmodel.expasy.org, accessed on 1 December 2024) and AlphaFold Server (https://alphafold.ebi.ac.uk/, accessed on 6 September 2024), and the protein was visualized using the Pymol bioinformatics program version 2.6 (LTS) (https://www.pymol.org/, accessed on 1 December 2024).

## 3. Results

### 3.1. Study Population

The study population of this investigation consisted of 111 patients with low birth weight, of which 47 were treated with growth hormone, and 65 did not receive treatment. Once the inclusion and exclusion criteria of the study were applied, the population was reduced to 28 patients: 14 patients treated with growth hormone (cases) and 14 patients not treated (controls). A comparative statistical analysis was performed between cases and controls regarding clinical manifestations (child of a mother with gestational diabetes, toxemia, arterial hypertension, gestational hemorrhage, threatened preterm delivery, threatened abortion, birth weight, gestations, gestational age, prenatal history, body mass index, growth hormone dose, waist circumference, total cholesterol, HDL cholesterol, LDL cholesterol, triglycerides, and glucose) and sociodemographic data (sex, maternal age at delivery, and exposure to tobacco), and differential genetic methylation profiles were performed on the 16 patients to evaluate the protective activity of growth hormone treatment in small-gestational-age children against obesity, during the 10-year follow-up of the study.

### 3.2. Collection of Clinical and Sociodemographic Data

The clinical and sociodemographic data of the 28 patients were analyzed, comparing the average values of the variables between cases and controls. The following results were found: 50% of the patients treated with growth hormone had an antecedent of threatened preterm delivery compared to 14.4% in the untreated group (*p* = 0.04). No differences were found in the other variables analyzed (Table 1).

**Figure 1:**
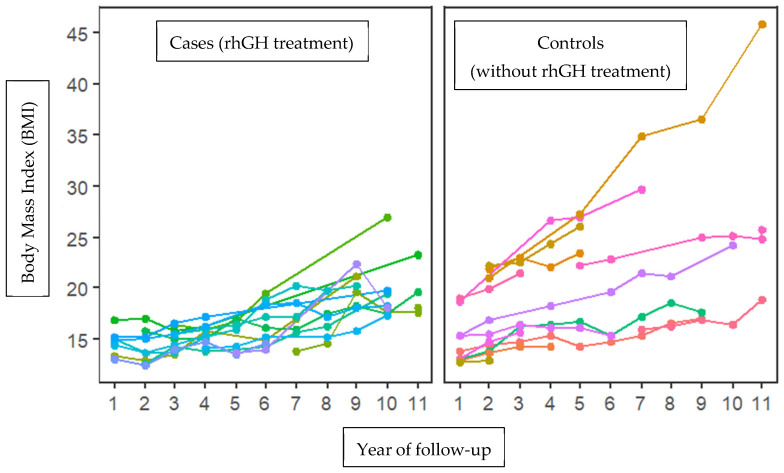
Increasing trend in body mass index (BMI) between cases (growth hormone treatment) and controls (without treatment with growth hormone) during 11 years of follow-up. A tendency towards increased BMI is observed in patients not treated with the growth hormone, with significant differences in times (2, 3, and 4). Each color corresponds to a patient’s follow-up for 10 years.

Additionally, the BMI behavior was analyzed between the case and control groups during the 11-year follow-up cohort of small gestational patients. Body mass index was higher in the group without treatment with growth hormone at time 2 (14.24 in the group with rhGH and 17.01 in the group without rhGH, with statistically significant differences (*p* = 0.035). This trend was also observed at time 3 (14.98 vs. 18.04; *p* = 0.049) and at time 4 (15.18 vs. 21.60; *p* = 0.009). No statistically significant differences were observed at other times (Figure 1).

**Figure 2:**
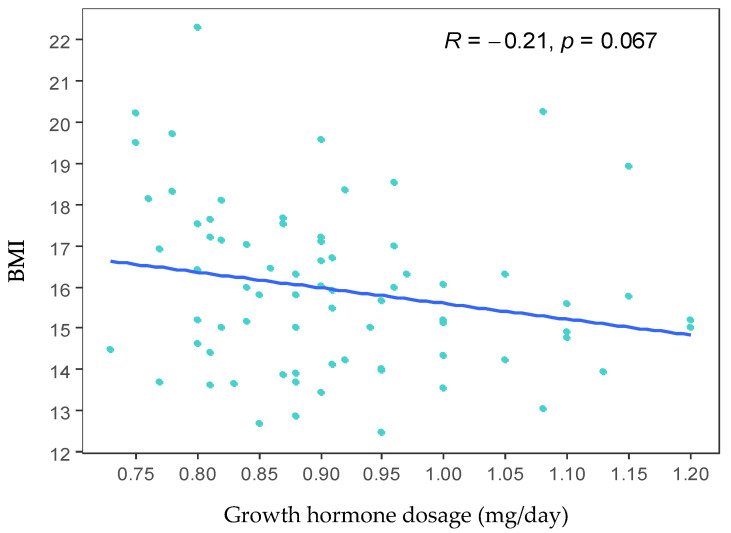
Scatter plot between growth hormone dose and body mass index (BMI). The graph shows a tendency towards a negative correlation between BMI and the growth hormone dose. However, this correlation is not statistically significant (*p* = 0.067).

Subsequently, the correlation between BMI and the dose of growth hormone used to treat the cases was evaluated. A tendency towards a negative correlation between BMI and the dose of growth hormone was observed: as the dose of growth hormone increases, the BMI decreases (R = −0.21). However, this correlation is not statistically significant (*p* = 0.067) (Figure 2).

### 3.3. Blood Biochemical Evaluation

The case and control groups were evaluated for average waist circumference, lipid profile (total cholesterol, HDL cholesterol, LDL cholesterol, and triglycerides), and peripheral blood glucose concentration. However, no statistically significant differences were found between the case and control groups. A tendency towards significance was only found in triglyceride values (*p* = 0.06) (Table 2).

### 3.4. DNA Sample Extraction

**Figure 3:**
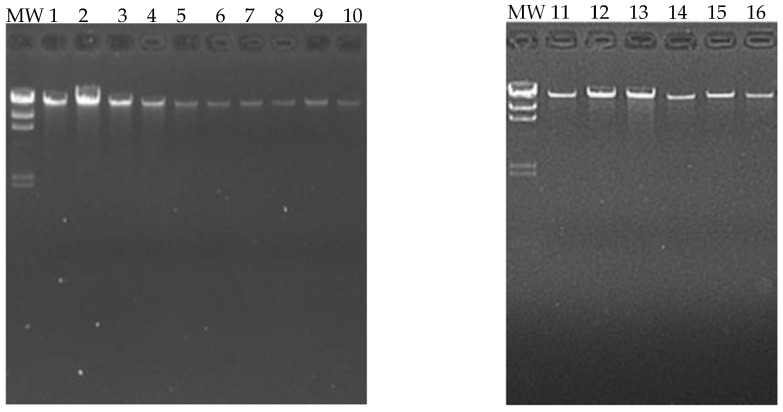
Extraction and quality analysis of DNA. The following figure shows a 1.5% agarose gel, on which the genetic material extracted from the 16 patients (eight cases and eight controls) can be visualized. MW (molecular weight marker, 1000 base pair scale).

Sixteen blood samples from patients (eight cases and eight controls) were chosen for the DNA extraction process. The quality of the samples was assessed using a 1.5% agarose gel, and it was found that all 16 samples had good genetic material integrity, and no degradation was observed (Figure 3).

### 3.5. Quantification and Evaluation of DNA Quality

The sixteen selected samples were evaluated for protein contamination using the ratio (260/280), finding a range of values between (1.81 and 2.08), highlighting the DNA’s purity. Additionally, the contamination with salts, alcohols, and phenols of the samples was evaluated using the ratio (260/230), finding a range of values between (1.81 and 2.14), confirming the DNA’s purity. Regarding the concentration of the samples, values between (0.58 and 3.34 ug of DNA) were obtained; these concentrations are sufficient to perform the epigenetic sequencing of the human methylation profile (Table 3).

### 3.6. Epigenetic Sequencing of Human Methylation Profiling

**Figure 4:**
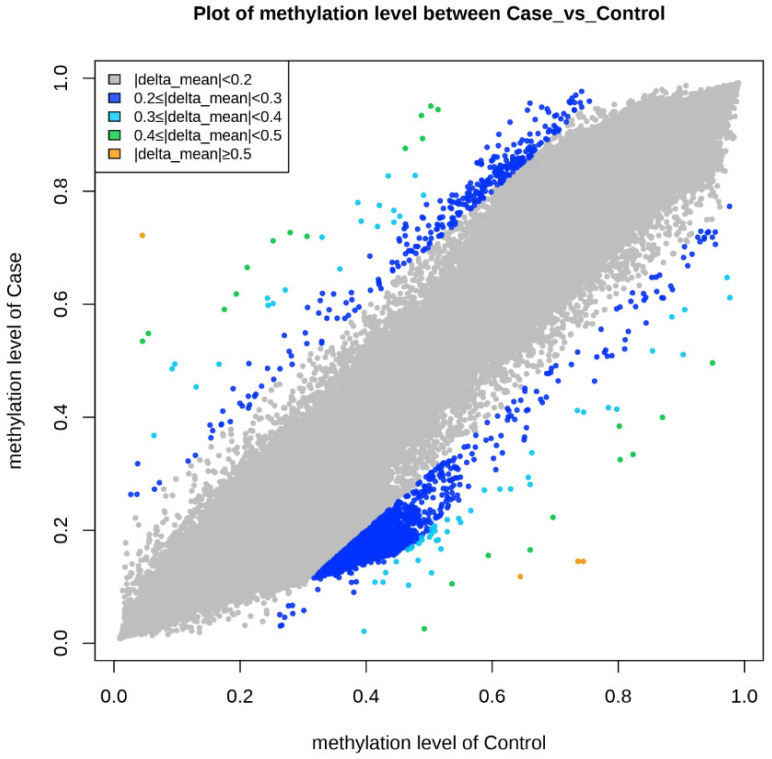
The graph shows the comparison of methylation levels between cases and controls. Each point on the graph represents a methylated region of the genome. The gray color represents delta mean values (<0.2) or methylated regions without differences in methylation levels, dark blue points are methylated regions with delta mean values (<0.3), light blue points are methylated regions with delta mean values (<0.4), green points are methylated regions with delta mean values (<0.5), and orange points are methylated regions with delta mean values (greater than or equal to 0.5).

**Figure 5:**
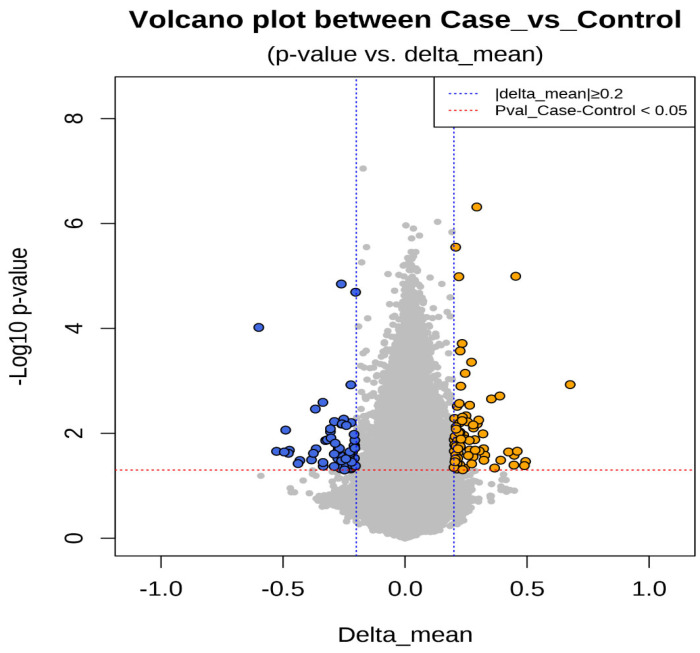
The figure shows a statistical analysis using the ***p***-value and mean delta values of the CpGs regions to filter out the methylated regions with the highest statistically significant differences between cases and controls. The results are graphed using a Volcano plot. The *p*-value of the CpGs regions is located on the *Y*-axis. The delta mean values of the CpG regions are located on the *X*-axis. Gray circles represent CpG regions without differences in methylation levels, blue circles represent CpG regions with decreased methylation values (hypomethylation), and yellow circles represent CpG regions with increased methylation values (hypermethylation).

**Figure 6:**
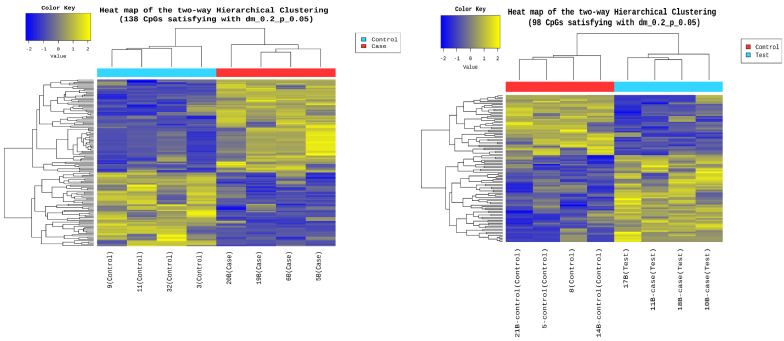
Heat map between the analyzed patients and the 138 differentially methylated CpG regions. The figure shows on the *Y*-axis the 138 CpG regions with statistically significant differences in the methylation level (delta-mean: 0.2, and *p* = 0.05). The analyzed patients, controls (blue rectangle), and cases (red rectangle) are shown on the *X*-axis. In the central part of the heat map, the number of times the methylation level increases or decreases is shown, with a decrease between (−2 and 0) (blue color) and an increase between (0 and +2) (yellow color).

Epigenetic sequencing of the human methylation profile was performed on 16 patients (8 cases and 8 controls) using the Infinium Methylation Epic v2.0 BeadChip methodology, which analyzes around 935,000 enhancer regions, gene bodies, promoters, and CpG islands. Figure 4 shows the methylation analysis of the 935,000 CpG regions analyzed in the genomes of the 16 patients. Many CpG regions had no differences in methylation patterns between cases and controls with delta mean values (<0.2) (gray dots). However, some CpG regions had subtle differences in methylation profiles with delta mean values between (0.3 and 0.4) (dark blue, light blue, and green dots), and even with statistically significant values greater than or equal to (0.5) (yellow dots) (Figure 4). An additional statistical analysis was performed using the *p*-value and mean delta values to filter out the methylated regions with the highest statistically significant differences between cases and controls. The results of this analysis are presented in Figure 5 using a Volcano plot; blue dots represent CpG regions with decreased methylation patterns, and yellow dots represent CpG regions with increased methylation patterns; 138 differentially methylated CpG regions were found. Figure 6 shows a heat map between the analyzed patients (cases and controls) and the 138 CpG regions that were found to be differentially methylated, with statistically significant differences in the methylation level (delta-mean ≥ 0.2 and *p* = 0.05). In the central part of the heat map, the number of folds in which the methylation level increases or decreases is shown (the values range from −2 to +2 times). Additionally, 24 hypermethylated genes (*MDGA1*, *HOXA5*, *LINC01168*, *ZFYVE19*, *ASAH1*, *MYH15*, *DNAJC17*, *PAMR1*, *MROCKI*, *CNDP2*, *CBY2*, *ZADH2*, *HOOK2*, *C9orf129*, *NXPH2*, *OSCP1*, *ZMIZ2*, *RUNX1*, *PTPRS*, *TEX26*, *EIF2A4K*, *MYO1F*, *C2orf69*, and *ZSCAN1*), and 21 hypomethylated genes (*C10orf71-AS1, ZDHHC13, RPL17, EMC4, RPRD2, OBSCN-AS1, ZNF714, MUC4, SUGT1P4, TRIM38, C3, SPON1, NGF-AS1, CCSER2, P2RX2, LOC284379, GGTA1, NLRP5, OR51A4, HLA-H*, and *TTLL8*) were identified, and the statistical values supporting the differential methylation patterns of these genes are shown in Table 4.

The biological function of hypermethylated and hypomethylated genes was determined by searching the GeneCards database (https://www.genecards.org/) [25]. The 24 hypermethylated genes have the following functions: The *MDGA1* gene encodes a glycosylphosphatidylinositol (GPI)-anchored cell surface glycoprotein that is predominantly expressed in the developing nervous system (https://www.genecards.org/cgi-bin/carddisp.pl?gene=MDGA1&keywords=MDGA1, accessed 25 October 2024). The *HOXA5* gene belongs to a class of transcription factors called homeobox genes. These are found in groups A, B, C, and D on different chromosomes. The expression of these proteins is regulated spatially and temporally during embryonic development (https://www.genecards.org/cgi-bin/carddisp.pl?gene=HOXA5&keywords=HOXA5, accessed 25 October 2024). The *LINC01168* gene (Long Intergenic Non-Protein Coding RNA 1168) is an RNA gene and is affiliated with the lncRNA class. The *ZFYVE19* gene enables phosphatidylinositol-3-phosphate binding activity. It participates in mitotic cytokinesis checkpoint signaling and negative regulation of cytokinesis. It is in the centrosome (https://www.genecards.org/cgi-bin/carddisp.pl?gene=LINC01168&keywords=LINC01168, accessed 25 October 2024). The *ASAH1* gene is a member of the acid ceramidase protein family. Processing of this pre-proprotein generates alpha and beta subunits that heterodimerize to form the mature lysosomal enzyme, which catalyzes the degradation of ceramide into sphingosine and free fatty acid (https://www.genecards.org/cgi-bin/carddisp.pl?gene=ASAH1&keywords=ASAH1, accessed 25 October 2024). The *MYH15* gene has several functions, including ATP binding activity, actin filament binding activity, and calmodulin (https://www.genecards.org/cgi-bin/carddisp.pl?gene=MYH15&keywords=MYH15, accessed 25 October 2024). The *DNAJC17* gene is a gene that allows RNA binding activity; it acts before or within the negative regulation of transcription by RNA polymerase II and toxin transport (https://www.genecards.org/cgi-bin/carddisp.pl?gene=DNAJC17&keywords=DNAJC17, accessed 25 October 2024). The *PAMR1* gene encodes a protein containing the peptidase domain; it also allows calcium ion binding activity and serine-type endopeptidase activity (https://www.genecards.org/cgi-bin/carddisp.pl?gene=PAMR1&keywords=PAMR1, accessed 25 October 2024). The *MROCKI* gene’s function is based on the CIS regulation of the cytokine promoter (https://www.genecards.org/cgi-bin/carddisp.pl?gene=MROCKI&keywords=MROCKI, accessed 25 October 2024). The *CNDP2* gene encodes for tissue carnosinase and peptidase A (https://www.genecards.org/cgi-bin/carddisp.pl?gene=CNDP2&keywords=CNDP2, accessed 25 October 2024). The *CBY2* gene enables identical protein binding activity. It is predicted to be in cytoplasmic vesicles (https://www.genecards.org/cgi-bin/carddisp.pl?gene=CBY2&keywords=CBY2, accessed 25 October 2024). The *ZADH2* gene is predicted to enable 13-prostaglandin reductase activity. It is predicted to be involved in the negative regulation of fat cell differentiation. It is predicted to be in the peroxisome. (https://www.genecards.org/cgi-bin/carddisp.pl?gene=PTGR3, accessed 25 October 2024). The *HOOK2* gene has microtubule-binding and C-terminal domains, which mediate binding to organelles (https://www.genecards.org/cgi-bin/carddisp.pl?gene=HOOK2&keywords=HOOK2, accessed 25 October 2024). The *C9orf129* gene is a coding gene with no known prior function. The *NXPH2* gene is predicted to enable signaling receptor binding activity. It is predicted to be in the extracellular region (https://www.genecards.org/cgi-bin/carddisp.pl?gene=NXPH2&keywords=NXPH2, accessed 25 October 2024). The *OSCP1* gene enables transmembrane transporter activity. It is involved in xenobiotic detoxification by transmembrane export across the plasma membrane. It is located in basal plasma membrane and cytoplasm (https://www.genecards.org/cgi-bin/carddisp.pl?gene=OSCP1&keywords=OSCP1, accessed 25 October 2024). The *ZMIZ2* gene is a member of the PIAS-like protein family that interacts with nuclear hormone receptors. It interacts with the androgen receptor (https://www.genecards.org/cgi-bin/carddisp.pl?gene=ZMIZ2&keywords=ZMIZ2, accessed 25 October 2024). The *RUNX1* gene encodes a heterodimeric transcription factor that binds to the central element of many enhancers and promoters (https://www.genecards.org/cgi-bin/carddisp.pl?gene=RUNX1&keywords=RUNX1, accessed 25 October 2024). The *PTPRA* gene is a coding gene with no known prior function (https://www.genecards.org/cgi-bin/carddisp.pl?gene=PTPRS&keywords=PTPRS, accessed 25 October 2024). The *TEX26* gene is characterized by being active in the cytoplasm (https://www.genecards.org/cgi-bin/carddisp.pl?gene=TEX26&keywords=TEX26, accessed 25 October 2024). The *EIF2A4K* gene encodes a member of the kinase family that phosphorylates the alpha subunit of eukaryotic translation initiation factor 2, resulting in the negative regulation of protein synthesis (https://www.genecards.org/cgi-bin/carddisp.pl?gene=TEX26&keywords=TEX26, accessed 25 October 2024). The *MYO1F* gene encodes an unconventional myosin that may be involved in intracellular movement (https://www.genecards.org/cgi-bin/carddisp.pl?gene=MYO1F&keywords=MYO1F, accessed 25 October 2024). The *C2orf69* gene is involved in oxidative phosphorylation (https://www.genecards.org/cgi-bin/carddisp.pl?gene=C2orf69&keywords=C2orf69, accessed 25 October 2024). The *ZSCAN1* gene is involved in the regulation of transcription by RNA polymerase II (https://www.genecards.org/cgi-bin/carddisp.pl?gene=ZSCAN1&keywords=ZSCAN1, accessed 25 October 2024).

**Figure 7:**
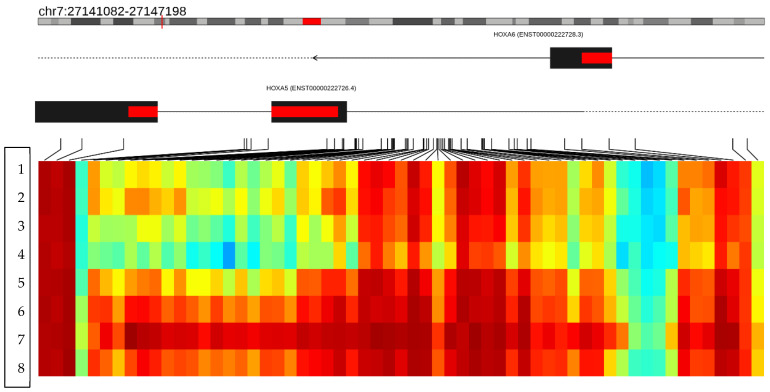
The figure shows a differentially methylated region located on the short arm of chromosome 7 (p15.2). The figure merges the neighboring CpGs that show a variation consistent with the hypermethylation status in the *HOXA5 gene*. The columns represent the CpG islands and the rows the patients analyzed. The first four rows show the methylation status of the *HOXA5* gene in the controls, and rows 5–8 represent the methylation status of the *HOXA5* gene in the cases. The heat map represents the methylation status of the CpG regions of the *HOXA5* gene; the red color represents the hypermethylated regions, and the blue color the hypomethylated regions, evidencing a hypermethylation state in the *HOXA5* gene in the cases. Red color indicates hypermethylated regions and light colors indicate hypomethylated regions.

The 21 hypomethylated genes have the following functions: The *C10orf71-AS1* gene (C10orf71 Antisense RNA 1) is an RNA gene and is affiliated with the lncRNA class (https://www.genecards.org/cgi-bin/carddisp.pl?gene=C10orf71-AS1&keywords=C10orf71-AS1, accessed 25 October 2024). The *OBSCN-AS1* gene (OBSCN Antisense RNA 1) is an RNA gene affiliated with the lncRNA class. (https://www.genecards.org/cgi-bin/carddisp.pl?gene=OBSCN-AS1&keywords=OBSCN-AS1, accessed 25 October 2024). The *NGF-AS1* gene (NGF Antisense RNA 1) is an RNA gene affiliated with the lncRNA class. Diseases associated with NGF-AS1 alterations include neuropathy, hereditary sensory and autonomic neuropathy type V, and autonomic neuropathy (https://www.genecards.org/cgi-bin/carddisp.pl?gene=NGF-AS1&keywords=NGF-AS1, accessed 25 October 2024). The *ZDHHC13* gene promotes magnesium ion transmembrane transporter activity and palmitoyl transferase activity. Furthermore, it is involved in the positive regulation of I-kappaB/NF-kappaB kinase signaling (https://www.genecards.org/cgi-bin/carddisp.pl?gene=ZDHHC13&keywords=ZDHHC13, accessed 25 October 2024). The *RPL17* gene encodes a ribosomal protein that is a component of the 60S subunit. The protein belongs to the L22P family of ribosomal proteins. It is in the cytoplasm. This gene has been referred to as rpL23 because the encoded protein shares amino acid identity with ribosomal protein L23 from Halobacterium marismortui; however, its official symbol is *RPL17*. As is typical for genes encoding ribosomal proteins, multiple processed pseudogenes of this gene are dispersed through the genome. Alternative splicing results in multiple transcript variants (https://www.genecards.org/cgi-bin/carddisp.pl?gene=RPL17&keywords=RPL17, accessed 25 October 2024). The *EMC4* gene contributes to membrane insertase activity. It is involved in protein insertion into ER membrane by stop-transfer membrane-anchor sequence and tail-anchored membrane protein insertion into the ER membrane. It is an integral component of the endoplasmic reticulum membrane. It is part of the EMC complex (https://www.genecards.org/cgi-bin/carddisp.pl?gene=EMC4&keywords=EMC4, accessed 25 October 2024). The *RPRD2* gene is involved with RNA polymerase II complex binding (https://www.genecards.org/cgi-bin/carddisp.pl?gene=RPRD2&keywords=RPRD2, accessed 25 October 2024). The *ZNF714* gene functions as a DNA-binding transcription factor, activating the specific binding of RNA polymerase II and the CIS regulatory region of RNA polymerase II (https://www.genecards.org/cgi-bin/carddisp.pl?gene=ZNF714&keywords=ZNF714, accessed 25 October 2024). The *MUC4* gene is known to be an integral membrane glycoprotein found on the cell surface (https://www.genecards.org/cgi-bin/carddisp.pl?gene=MUC4&keywords=MUC4, accessed 25 October 2024). The *TRIM38* gene encodes a member of the tripartite motif (TRIM) family. The encoded protein contains a RING-type zinc finger, B box-type zinc finger, and SPRY domain. The function of this protein has not been identified. A pseudogene of this gene is located on the long arm of chromosome 4 (https://www.genecards.org/cgi-bin/carddisp.pl?gene=TRIM38&keywords=TRIM38, accessed 25 October 2024). The *C3* gene encodes a protein known as anaphylatoxin C3a, which modulates inflammation and has antimicrobial activity (https://www.genecards.org/cgi-bin/carddisp.pl?gene=C3&keywords=C3, accessed 25 October 2024). The *SPON1* gene function is related to the structure of the extracellular matrix and cell adhesion. It also acts within the positive regulation of protein binding and processing activity (https://www.genecards.org/cgi-bin/carddisp.pl?gene=SPON1&keywords=SPON1, accessed 25 October 2024). The *CCSER2* gene allows binding to microtubules; it is in the cytoplasm (https://www.genecards.org/cgi-bin/carddisp.pl?gene=CCSER2&keywords=CCSER2, accessed 25 October 2024). The *P2RX2* gene belongs to the ATP purine receptor family, which functions as a ligand-gated ion channel, and ATP binding mediates synaptic transmission between neurons (https://www.genecards.org/cgi-bin/carddisp.pl?gene=P2RX2&keywords=P2RX2, accessed 25 October 2024). The *GGTA1* gene is thought to encode a truncated, non-enzymatic form of the GGTA1 protein that lacks the C-terminal catalytic domain (https://www.genecards.org/cgi-bin/carddisp.pl?gene=GGTA1&keywords=GGTA1, accessed 25 October 2024). The *NLRP5* gene, encoded by this gene, belongs to the NALP protein family. Members of the NALP protein family typically contain a NACHT domain, a NACHT-associated domain (NAD), a C-terminal leucine-rich repeat (LRR) region, and an N-terminal pyrin domain (PYD). Expression of this gene is restricted to the oocyte. A mouse gene that encodes a maternal oocyte protein, like this encoded protein, is required for normal early embryogenesis (https://www.genecards.org/cgi-bin/carddisp.pl?gene=NLRP5&keywords=NLRP5, accessed 25 October 2024). In the *OR51A4* gene, olfactory receptors interact with odorant molecules in the nose to initiate a neuronal response that triggers the perception of a smell. The olfactory receptor proteins are members of a large family of G-protein-coupled receptors (GPCRs) arising from single coding-exon genes. Olfactory receptors share a seven-transmembrane-domain structure with many neurotransmitter and hormone receptors and are responsible for the recognition and G protein-mediated transduction of odorant signals. The olfactory receptor gene family is the largest in the genome. (https://www.genecards.org/cgi-bin/carddisp.pl?gene=OR51A4&keywords=OR51A4, accessed 25 October 2024). The *HLA-H* gene, a major histocompatibility complex gene, represents a transcribed pseudogene, possibly derived from HLA-A. This gene displays extensive variation (https://www.genecards.org/cgi-bin/carddisp.pl?gene=HLA-H&keywords=HLA-H, accessed 25 October 2024). The *TTLL8* gene is predicted to enable protein–glycine ligase activity initiation. It is predicted to be involved in protein polyalkylation. Predicted to act upstream of or within cilium assembly. Predicted to be in axoneme and microtubule cytoskeleton (https://www.genecards.org/cgi-bin/carddisp.pl?gene=TTLL8&keywords=TTLL8, accessed 25 October 2024). Finally, a differentially hypermethylated region was found on the short arm of chromosome 7 at position (p15.2) between the nucleotides (27141082–27147198), corresponding to the region that codes for the *HOXA5* gene. In this region, clear hypermethylation is observed along the gene, with approximately 59 methylated CpG sites in the genomes of patients treated with growth hormone (cases) (Figure 7).

### 3.7. Bioinformatic Prediction of Protein Interactions

**Figure 8:**
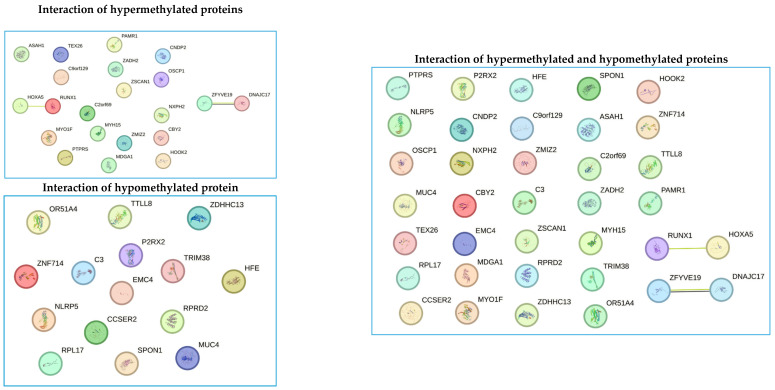
Bioinformatics detection of interactions between the group of hypermethylated proteins, the group of hypomethylated proteins, and all proteins with differential methylation profiles between cases and controls. Each circle represents a gene encoding a protein with differential methylation patterns (hypermethylated or hypomethylated). The lines between points represent the interaction between the different proteins. The figure shows the interaction between (*RUNX1* and *HOXA5*), and (*ZFYVE19* and *DNAJC17*). These interacting proteins were found to be hypermethylated. The yellow interaction lines mean that the interaction was detected by text mining, and the purple lines mean that the data come from co-expression assays.

Protein interaction analysis revealed interactions between proteins encoded by genes (*RUNX1* and *HOXA5*), and (*ZFYVE19* and *DNAJC17*). Both interactions correspond to proteins that are in the group of hypermethylated proteins. These interactions indicate a synergy between proteins and are related to a hypermethylation genotype. On the other hand, interactions between hypomethylated proteins could not be predicted. Additionally, when predicting interactions between the groups of hypermethylated and hypomethylated proteins, no interaction between them could be predicted, and the two interactions between hypermethylated proteins were maintained (Figure 8).

### 3.8. Structural Modeling and Functional Bioinformatics Analysis

**Figure 9:**
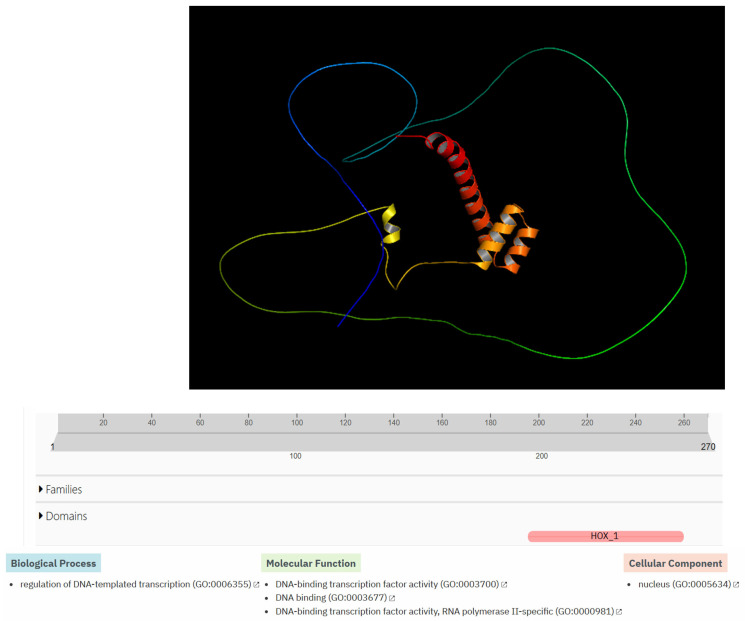
Structural modeling and functional bioinformatics analysis of the HOX-A5 protein. The top of the figure shows bioinformatics prediction of the three-dimensional structure of the Homeobox protein Hox-A5 using AlphaFold3. The middle part of the figure shows the predicted domain (HOX_1), which is highlighted in pink. The bottom of the figure shows the biological processes, molecular function, and cellular components related to the HOX-A5 protein, which were predicted using the InterProScan bioinformatics program. The bottom of the figure shows one biological process, three molecular functions, and one cellular component related to the protein.

The Hox-A5 protein is made up of 270 amino acids. It has a simple three-dimensional structure with four alpha helices inside the protein; a long chain of amino acids surrounds the internal part of the protein without a defined secondary structure. At the functional level, it has a HOX_1 domain. At the level of biological processes, it is related to the regulation of DNA template transcription GO:0006355. Additionally, it is related to three molecular functions (DNA-binding transcription factor activity GO:0003700, DNA binding GO:0003677, DNA-binding transcription factor activity—RNA polymerase II-specific GO:0000981). In addition, it is predicted that this protein is in the cell nucleus (nucleus GO:0005634) (Figure 9).

## 4. Discussion

Overweight and obesity in childhood and adolescence obesity are among the most significant public health challenges of our time due to their widespread prevalence, associated morbidity, and mortality [26]. Obesity is a polygenic and multifactorial condition, in which various environmental, genetic, and epigenetic factors interact to contribute to its development [27]. This condition is characterized by a complex metabolic imbalance that leads to significant changes in numerous biological processes, including the regulation of the central and peripheral nervous systems, energy balance, glucose homeostasis, lipids, and adipose tissue, along with their interactions [28]. In this study, we investigated a cohort of small-for-gestational-age (SGA) newborns to evaluate whether growth hormone treatment during infancy serves as a protective factor against the onset of obesity in adolescence. Furthermore, we explored changes in gene methylation patterns associated with this treatment to identify potential epigenetic biomarkers for obesity in the future.

The clinical and sociodemographic data of 28 patients were analyzed, comparing the average values of various variables between the cases and controls. The results showed that 50% of the patients treated with growth hormone had a history of threatened preterm delivery, compared to only 14.4% in the untreated group (*p* = 0.04), indicating a significant association between threatened preterm birth and small-gestational-age children. No differences were found in the other analyzed variables (see Table 1). However, body mass index (BMI) was significantly higher in the untreated group at time 2, with average values of 14.24 for the growth hormone (rhGH) group and 17.01 for the untreated group (*p* = 0.035). This trend continued at time 3 (14.98 vs. 18.04; *p* = 0.049) and at time 4 (15.18 vs. 21.60; *p* = 0.009). No statistically significant differences were observed at other times (see Figure 1), which suggests that growth hormone treatment in small-gestational-age children may be linked to a decrease in body mass index during adolescence. Recent studies have found that rhGH treatment leads to favorable changes in body composition, including an increase in lean mass and a decrease in fat mass [23,24].

Subsequently, the correlation between BMI and the dose of growth hormone administered for treatment was evaluated. A negative correlation was found higher doses of growth hormone are associated with a decrease in BMI (R = −0.21). However, this relationship only approached significance (*p* = 0.067), and the data were quite scattered (see Figure 2). Recent studies have reported a decrease in BMI among children treated with growth hormone for up to six months [29] or two years [30]; after this period, these children tend to experience an increase in BMI. Additionally, DNA methylation is increasingly recognized as a promising biomarker for obesity-related traits. Quantitatively, these methylation markers account for a significant portion of the variance in obesity-related traits. For instance, one study identified 94 CpG sites linked to BMI and 49 CpG sites correlated with waist circumference [31].

The case and control groups were assessed for average waist circumference, lipid profile (including total cholesterol, HDL cholesterol, LDL cholesterol, and triglycerides), and peripheral blood glucose concentration. However, no statistically significant differences were observed between the two groups. A tendency towards significance was noted for triglyceride levels (*p* = 0.06) (see Table 2). Some studies have indicated that treatment with growth hormone may reduce serum triglyceride concentrations [32]. Additionally, the treatment has been shown to be effective in improving normal values of total cholesterol (TC), non-HDL cholesterol, and LDL cholesterol levels [33].

Sixteen blood samples from patients, consisting of eight case subjects and eight control subjects, were selected for the DNA extraction process. The quality of the samples was assessed using a 1.5% agarose gel, revealing that all 16 samples maintained good genetic material integrity without any observed degradation (see Figure 3). These findings indicate that the blood samples were extracted properly and are suitable for epigenomic sequencing. The samples were further evaluated for protein contamination using a 260/280 ratio, which yielded values ranging from 1.81 to 2.08, indicating high DNA purity. Additionally, contamination with salts, alcohol, and phenols was assessed using a 260/230 ratio, resulting in values between 1.81 and 2.14, further confirming the DNA’s purity. The concentration of DNA in the samples ranged from 0.58 to 3.34 µg, which is adequate for performing the epigenetic sequencing of the human methylation profile. The human methylation profile was sequenced across the 16 patients (8 cases and 8 controls). Figure 4 displays the methylation analysis of 935,000 CpG regions in the genomes of these patients. While many CpG regions showed no significant differences in methylation patterns between cases and controls, with delta mean values less than 0.2, several regions exhibited subtle differences, with delta mean values ranging from 0.3 to 0.5, and some had statistically significant values of 0.5 or greater (see Figure 4). A subsequent statistical analysis, utilizing *p*-values and mean delta values, was conducted to identify methylated regions with the most significant differences between cases and controls. The outcomes of this analysis are illustrated in Figure 5, using a Volcano plot. As a result, 138 CpG regions were found to have statistically significant differences between the cases and controls. Figure 6 presents a heat map of these analyzed patients (both cases and controls) alongside the 138 differentially methylated CpG regions, which demonstrated significant differences in methylation levels (delta mean: 0.2, *p* = 0.05). While most genomic CpG regions displayed similar methylation patterns between cases and controls, 138 genomic regions showed statistically significant differences. The differential methylation analysis specifically focused on coding regions, identifying 24 hypermethylated genes (*MDGA1, HOXA5, LINC01168, ZFYVE19, ASAH1, MYH15, DNAJC17, PAMR1, MROCKI, CNDP2, CBY2, ZADH2, HOOK2, C9orf129, NXPH2, OSCP1, ZMIZ2, RUNX1, PTPRS, TEX26, EIF2A4K, MYO1F, C2orf69*, and *ZSCAN1*) and 21 hypomethylated genes (*C10orf71-AS1, ZDHHC13, RPL17, EMC4, RPRD2, OB-SCN-AS1, ZNF714, MUC4, SUGT1P4, TRIM38, C3, SPON1, NGF-AS1, CCSER2, P2RX2, LOC284379, GGTA1, NLRP5, OR51A4, HLA-H*, and *TTLL8*). Statistical values for each gene are provided in Table 4. Among the hypermethylated genes, two that are related to obesity are noteworthy: *ASAH1* and *ZADH2*. The *ASAH1* gene belongs to the acid ceramidase protein family. Processing of this pre-proprotein generates alpha and beta subunits that heterodimerize to form the mature lysosomal enzyme, which catalyzes the degradation of ceramide into sphingosine and free fatty acid. The *ZADH2* gene is predicted to enable 13-prostaglandin reductase activity and is thought to be involved in the negative regulation of fat cell differentiation; it is believed to be in the peroxisome. Additionally, the *OBSCN-AS1* gene (OBSCN Antisense RNA 1), an RNA gene affiliated with the lncRNA class, was found to be hypomethylated in both sequencing assays of the methylation patterns. These trends towards significance suggest the need for new research with a larger population size and more economic resources that allow us to evaluate the sociodemographic, clinical, and epigenomic characteristics of a larger population.

Several studies have demonstrated that epigenetic mechanisms play a role in the onset and progression of obesity and its metabolic complications. These mechanisms are influenced by environmental and individual factors, which can be categorized into two main types: nutritional and physiological [34]. Specific genes have been identified whose methylation patterns are associated with obesity, type 2 diabetes, and metabolic syndrome. These genes are involved in various signaling pathways, including those related to insulin resistance and secretion (such as *ATM, PTPRN2, PSMD10*, and *NSF*), adipogenesis (notably *SLC25A24* and *PAX8*), inflammatory processes (including *TNFRSF8* and *SLIT3*), and mitochondrial functions (like *PM20D1* and *LCLAT1*). Furthermore, studies examining the methylation patterns of CpG islands in obese versus non-obese individuals have found differential methylation of genes such as *NRF1*, *ADR1B*, and *PTPRN2*, highlighting their potential as biomarkers for obesity risk [35,36].

Additionally, we identified a differentially methylated region associated with the *HOXA5* gene. Within this region, nearly 59 methylated CpG sites were observed in the genomes of patients treated with growth hormone (cases), indicating significant hypermethylation. *HOX* genes are members of the Homeobox transcription factor superfamily, which play a critical role in embryonic development, hematopoiesis, and tumorigenesis [37]. The *HOX* gene family consists of 39 genes clustered in four chromosomal regions: *HOXA*, *HOXB*, *HOXC*, and *HOXD* [38]. These genes regulate tissue-specific gene expression, and any mutations or dysregulation can lead to developmental abnormalities and aberrant phenotypes in humans [39]. Recent scientific evidence shows that the *HOXA5* gene is highly expressed in adipose tissue and actively regulates adipocyte biology and body fat distribution [40]. Studies by Parrillo et al. have linked *HOXA5* expression to variations in adiposity levels and body fat distribution patterns [41].

Moreover, weight loss in obese individuals has been associated with the positive regulation of the *HOXA5* gene in subcutaneous adipose tissue. Methylation events complicate the regulation of the *HOXA5* gene in relation to metabolic diseases [42]. Increased methylation of the gene’s promoter region has been shown to reduce its expression in the preadipocytes of individuals with hypertrophic obesity [43]. Changes in methylation could also serve as a biomarker for predicting the risk of obesity and type 2 diabetes. Moreover, individuals with a family history of type 2 diabetes (T2D) and hypertrophic obesity exhibit elevated methylation levels of the *HOXA5* gene [44], which correlates with larger fat cell sizes and a higher BMI [43]. This suggests that the methylation profile of *HOXA5* may indicate levels of adiposity. In our findings, the *HOXA5* gene in the control patient group was hypermethylated, favoring its transcription and translation, thereby acting as a protective factor against the development of obesity in patients treated with growth hormone.

Bioinformatics prediction of protein–protein interactions is crucial for establishing processes such as signal transduction, protein complex formation, protein transport, and the successive interaction of proteins within metabolic pathways [45,46,47,48]. Understanding these processes allows us to delineate a cell’s metabolic pathways and interactome, leading to insights into the functionalities and synchronization of various biological processes within a cell. Additionally, this knowledge aids in studying the genesis and progression of numerous diseases [45,46,47,48]. In our study, we identified two interactions involving four proteins: *RUNX1* and *HOXA5*, as well as *ZFYVE19* and *DNAJC17*. However, experimental studies are necessary to validate the role of these interactions in obesity development. Once verified through experimentation, these four proteins could become valuable biomarkers for monitoring obesity. Among the genes analyzed, *HOXA5* was the most hypermethylated in patients treated with growth hormone, making it a strong candidate for a biomarker in the obesity process. Therefore, it is crucial to understand its structure, functional domains, biological processes, molecular functions, and cellular compartments using bioinformatics tools (Figure 9). Based on the information obtained through bioinformatics tools, we could speculate that hypermethylation of the gene that encodes the Hox-A5 protein could affect its function as a transcriptional factor, causing abnormalities in the biology of adipocytes and body fat distribution. However, experimental studies are needed to confirm the true role of this protein in the development of obesity.

Long non-coding RNAs (lncRNAs) are RNA molecules that regulate gene expression without being translated into proteins [49]. These RNAs can interact with DNA, RNA, proteins, and other non-coding RNA molecules to modulate gene expression [49]. They occupy a significant portion of the genomes of complex organisms and are involved in cell differentiation and development in both animals and plants [49]. Additionally, lncRNAs have been associated with various physiological processes, including cholesterol biosynthesis [50,51], growth hormone and prolactin production [52], and glucose metabolism [53,54]. Given their roles, lncRNAs appear to be important in processes related to obesity. In our study, we identified hypermethylated lncRNA (LINC01168) and hypomethylated lncRNAs (*NGF-AS1*, *OBSCN-AS1*, and *C10orf71-AS1*) in patients treated with growth hormone. This finding suggests that these lncRNAs may be involved in the genetic regulation of obesity-related processes. In the future, lncRNAs could serve as potential biomarkers for diagnosing and prognosing obesity.

The above findings have important clinical implications, as they suggest the protective role of growth hormone in preventing the development of obesity in adolescents who were small-for-gestational-age (SGA) newborns and postulate the *HOXA5* gene as a possible biomarker to help predict the early development of obesity. However, new studies that analyze a larger population and explore the sociodemographic, clinical, and epigenomic aspects are necessary to corroborate the findings of this research.

## 5. Conclusions

In this research, we evaluated the impact of recombinant human growth hormone (rhGH) on genetic methylation patterns and its relationship with body composition in small-for -gestational-age (SGA) newborns. We found that children who did not receive growth hormone treatment had higher body mass index (BMI) values. Conversely, higher doses of growth hormone treatment were associated with a reduction in BMI. Additionally, growth hormone use correlated with decreased triglyceride levels in the blood. We also identified hypermethylated and hypomethylated genes related to obesogenic processes in the cases studied, which may serve as potential biomarkers for obesity after further experimental verification. These findings suggest that administering growth hormone to SGA newborns could serve as a protective factor against the development of obesity in adolescence. The main limitations of this study were having a small cohort of patients and few economic resources to study the clinical, sociodemographic, and epigenetic characteristics of a larger population.

## Figures and Tables

**Table 1:** Statistical analysis between cases and controls of clinical and sociodemographic data. The table shows the average values of each variable and the *p*-value when comparing cases and controls. The meaning of the acronyms used in the table is described below: Son of a Mother with Gestational Diabetes (SMGD), High Blood Pressure (HBP), gestational hemorrhage (GH), Threat of Preterm Birth (TPB), Threat of Abortion (TA), Maternal Age at Delivery (MAD), and Standard Deviation (SD).

Variable	Category	Growth Hormone				*p*
		Yes		No		
		n	%	n	%	
Sex	Female	4	28.6	10	71.4	
	Male	10	71.4	4	28.6	
SMGD		1	7.70	1	7.70	0.95
Tobacco exposure		1	7.10	0	0.00	0.30
Toxemia		1	7.10	2	14.3	0.54
HBP		1	7.10	1	7.10	1.00
GH		2	14.3	1	7.10	0.54
TPB		7	50.0	2	14.3	0.04
TA		3	21.4	1	7.10	0.28
		Mean	SD	Mean	SD	*p*
Birth weight		1921.14	536.3	1949.21	483.21	0.88
MAD		28	7.33	27.71	4.76	0.90
Pregnancies		2.15	1.23	1.64	0.74	0.20
Gestational age		33.5	3.79	34.57	2.31	0.37
Prenatal history		6.57	4.01	7.78	1.05	0.29

**Table 2:** Average waist circumference values, lipid profile, and blood glucose concentration in the case group (treated with growth hormone) and controls (without treatment with growth hormone). The meaning of the acronyms used in the table is described below: Standard Deviation (DS).

Variable	Growth Hormone				*p*
	Yes		No		
	Mean	SD	Mean	SD	
Waist circumference (cm)	68.76	9.01	73.66	19.21	0.39
Total cholesterol (mg/dL)	156.92	17.3	155.42	36.55	0.89
HDL cholesterol (mg/dL)	53.66	13.1	46.43	9.12	0.1
LDL cholesterol (mg/dL)	94.92	16.55	97.02	33.93	0.83
Triglycerides (mg/dL)	86.14	36.44	139.57	95.16	0.06
Glucose (mg/dL)	91.42	7.42	89.92	9.23	0.64

**Table 3:** The sixteen samples used to perform DNA extraction and their purity values, concentration, and final volume.

#	Sample Name	Purity (260/280)	Purity (260/230)	Concentration (ng/μL)	Volume (μL)	Total Amount (μg)
1	11 (Control)	1.85	1.81	82.23	25	2.06
2	32 (Control)	1.87	1.99	104.33	14	1.46
3	9 (Control)	1.91	1.85	81	30	2.43
4	3 (Control)	1.87	1.97	42.1	20	0.84
5	8 (Control)	1.91	1.85	22.59	30	0.68
6	14B (Control)	2.02	2.1	28.27	30	0.85
7	21B (Control)	1.98	2.14	44.77	30	1.34
8	5 (Control)	1.94	1.86	46.78	30	1.4
9	20B (Case)	1.81	1.95	13.86	175	2.42
10	6B (Case)	1.84	1.85	14.91	180	2.68
11	5B (Case)	1.91	1.91	13.12	180	2.36
12	19B (Case)	1.82	1.81	18.57	180	3.34
13	17B (Case)	1.85	1.86	13.31	185	2.46
14	18B (Case)	2.08	2.04	23.09	30	0.69
15	10B (Case)	2.05	2.02	21.75	30	0.65
16	11B (Case)	2.01	1.86	19.21	30	0.58

**Table 4:** The table shows the names of the genes that were found to be hypermethylated and hypomethylated, the NCBI accession number of the gene, the mean methylation value of cases and controls, the delta mean value, *p*-value, the fold change, and the odds ratio.

Gene Name	Gene Accession	Control Mean	Case Mean	Delta Mean	*p*-Value	Fold Change	Odds Ratio	Methylation Status
*MDGA1*	NM_153487.4	0.4352	0.8272	0.3920	0.0325	2.2004	7.6790	Hypermethylated
*HOXA5*	NM_019102.4	0.4181	0.7375	0.3195	0.0102	1.7870	4.4147	Hypermethylated
*LINC01168*	NR_046231.1	0.4603	0.7336	0.2733	0.0383	1.5631	3.7230	Hypermethylated
*ZFYVE19*	NM_032850.5	0.5352	0.7693	0.2340	0.0002	1.4404	2.9114	Hypermethylated
*ASAH1*	NR_125429.1	0.4673	0.6945	0.2271	0.0182	1.4777	2.7093	Hypermethylated
*MYH15*	NM_014981.3	0.5901	0.8163	0.2261	0.0003	1.3881	3.0815	Hypermethylated
*DNAJC17*	NM_018163.3	0.2342	0.4550	0.2208	0.0000	1.9411	2.7305	Hypermethylated
*PAMR1*	NM_015430.4	0.5031	0.7166	0.2135	0.0373	1.4697	2.4634	Hypermethylated
*MROCKI*	NR_038863.2	0.5322	0.7417	0.2095	0.0083	1.4047	2.5660	Hypermethylated
*CNDP2*	NM_001168499.2	0.4905	0.6950	0.2044	0.0349	1.4147	2.5076	Hypermethylated
*CBY2*	NM_001286342.2	0.2132	0.4163	0.2031	0.0052	2.2042	2.9562	Hypermethylated
*ZADH2*	NM_001306093.1	0.2021	0.4381	0.2360	0.023839	2.1549	3.1290	Hypermethylated
*HOOK2*	NM_001100176.2	0.1994	0.6461	0.4467	0.021309	4.5404	12.282	Hypermethylated
*C9orf129*	NR_166069.1	0.0545	0.2682	0.2137	0.042371	3.6805	4.8420	Hypermethylated
*NXPH2*	NM_007226.3	0.3943	0.6575	0.2632	0.025904	1.7214	3.1292	Hypermethylated
*OSCP1*	NM_001330493.2	0.2899	0.5103	0.2203	0.003039	1.8052	2.6168	Hypermethylated
*ZMIZ2*	NM_001300959.2	0.4532	0.7045	0.2513	0.027977	1.5689	3.1253	Hypermethylated
*RUNX1*	NM_001001890.3	0.2143	0.4373	0.2229	0.043655	2.3259	3.2752	Hypermethylated
*PTPRS*	NM_001394011.1	0.5526	0.7648	0.2121	0.0000745	1.3850	2.6442	Hypermethylated
*TEX26*	NM_001353390.2	0.4745	0.7073	0.2327	0.026512	1.5207	2.7487	Hypermethylated
*EIF2A4K*	NM_001013703.4	0.5625	0.7739	0.2114	0.044890	1.3523	3.1282	Hypermethylated
*MYO1F*	NM_001348355.2	0.6045	0.8542	0.2497	0.013192	1.4221	5.1808	Hypermethylated
*C2orf69*	NM_153689.6	0.4277	0.6855	0.2578	0.041214	1.6343	3.3052	Hypermethylated
*ZSCAN1*	NM_182572.4	0.2962	0.5344	0.2382	0.033339	1.9793	2.9281	Hypermethylated
*C10orf71*	NR_108038.1	0.5094	0.3065	−0.2029	0.0418	−1.6093	0.4233	Hypomethylated
*ZDHHC13*	NM_001001483.3	0.6519	0.4462	−0.2057	0.0136	−1.4886	0.4246	Hypomethylated
*RPL17*	NM_001199342.3	0.5739	0.3681	−0.2058	0.0192	−1.6226	0.4230	Hypomethylated
*EMC4*	NM_001286420.2	0.7009	0.4912	−0.2097	0.0387	−1.4272	0.3798	Hypomethylated
*RPRD2*	NM_001387114.1	0.9296	0.7105	−0.2190	0.0354	−1.3311	0.2035	Hypomethylated
*OBSCN*	NM_001098623.2	0.5200	0.3004	−0.2195	0.0447	−1.8213	0.3751	Hypomethylated
*ZNF714*	NM_182515.4	0.5595	0.3199	−0.2395	0.0459	−1.7464	0.3589	Hypomethylated
*MUC4*	NM_004532.6_8	0.7046	0.4643	−0.2403	0.0422	−1.5140	0.3101	Hypomethylated
*SUGT1P4*	NR_036526.1	0.8832	0.6321	−0.2510	0.0054	−1.4048	0.2032	Hypomethylated
*TRIM38*	NM_006355.5	0.7799	0.5188	−0.2611	0.0000	−1.5043	0.3026	Hypomethylated
*C3*	NM_000064.4	0.4268	0.1082	−0.3186	0.0133	−3.5811	0.1735	Hypomethylated
*SPON1*	NM_006108.4	0.4823	0.1468	−0.3355	0.0424	−2.8404	0.1901	Hypomethylated
*NGF-AS1*	NR_157569.1	0.5362	0.1052	−0.4309	0.0330	−4.1430	0.1059	Hypomethylated
*CCSER2*	NM_001284243.2	0.6073	0.2451	−0.3622	0.0145040	−2.51360	0.1880	Hypomethylated
*P2RX2*	NM_174872.3	0.7155	0.5131	−0.2023	0.0307259	−1.37424	0.3882	Hypomethylated
*LOC284379*	NR_002938.3	0.5250	0.285	−0.2393	0.0289216	−1.77223	0.3563	Hypomethylated
*GGTA1*	NM_001382584.1	0.9308	0.6773	−0.2535	0.0320150	−1.40730	0.2004	Hypomethylated
*NLRP5*	NM_153447.4	0.7447	0.5068	−0.2378	0.0109644	−1.49354	0.3446	Hypomethylated
*OR51A4*	NM_001005329.2	0.4572	0.1365	−0.3206	0.0158866	−6.11715	0.1048	Hypomethylated
*HLA-H*	NR_001434.4_8	0.5794	0.3483	−0.2310	0.0098659	−1.65606	0.3809	Hypomethylated
*TTLL8*	NM_001350317.2	0.9795	0.7516	−0.2278	0.0008761	−1.308043	0.0649	Hypomethylated

## Data Availability

The raw data presented in this study are available on request from the corresponding author. The data are not publicly available due to ethical restrictions.
